# Genome-scale investigation of olfactory system spatial heterogeneity

**DOI:** 10.1371/journal.pone.0178087

**Published:** 2017-05-24

**Authors:** Torben Noto, Derrick Barnagian, Jason B. Castro

**Affiliations:** 1 Department of Cognitive Science, University of California San Diego, La Jolla, California, United States of America; 2 Department of Brain and Cognitive Sciences, MIT, Cambridge, Massachusetts, United States of America; 3 Neuroscience Program, Bates College, Lewiston, Maine, United States of America; Duke University, UNITED STATES

## Abstract

The early olfactory system is organized in parallel, with numerous, specialized subsystems established by the modular and topographic projections of sensory inputs. While these anatomical sub-systems are in many cases demarcated by well-known marker genes, we stand to learn considerably more about their possible functional specializations from comprehensive, genome-scale descriptions of their molecular anatomy. Here, we leverage the resources of the Allen Brain Atlas (ABA)—a spatially registered compendium of gene expression for the mouse brain—to investigate the early olfactory system’s genomic anatomy. We cluster thousands of genes across thousands of voxels in the ABA to derive several novel parcellations of the olfactory system, and concomitantly discover novel sets of enriched, subregion-specific genes that can serve as a starting point for future inquiry.

## Introduction

A major challenge in neuroanatomy is to partition the brain “at its joints” [1], identifying its minimal sub-systems and their potential heirarchical relationships [2]. While classical histology is essential for this endeavor, it may also be limited in some cases, owing to its unavoidable use of ad-hoc differentiating criteria. Even when discrete subregions can be demarcated by differences in the abundance and/or co-expression of specific molecules, these typically represent only a small and idiosyncratic subset of the molecules that collectively support excitability, synaptic communication, plasticity, neuromodulation, and the developmental specification of neural circuits.

With the advent of accessible, comprehensive data resources such as the Allen Brain Atlas (ABA) [3], these approaches can be complemented by data-driven frameworks for studying the brain’s molecular architecture. Because the gene-expression maps of the ABA are panoramic, dense (cataloging the entire mammalian genome), and quantitative, one can develop genomic definitions of brain areas that capture spatial correlations across many genes [3,4]. Indeed, such approaches have been successfully used to recapitulate the brain’s major phylogenetic subdivisions, and to map the more granular “genomic anatomy” of the hippocampus[5–8] and neocortex [4,9].

Here, we extend similar methods to investigate the genomic anatomy of the early olfactory system, including the olfactory bulb (OB)—the first central structure in the ascending olfactory system—and its more central cortical targets. As a model anatomical system, the OB has several virtues, the principal of which is the well-described molecular topography and modularity of its sensory inputs. Briefly, the bulb can be partitioned into several non-overlapping subregions, defined by both the projection patterns of molecularly labeled inputs [10–14], and in some cases, the contribution of these regions to specific behaviors or sets of behaviors (e.g. conditioned vs. innate odor responses [14]). At the same time, many basic questions on the olfactory system’s organization and substructure persist, highlighting a potentially important role for unbiased genomic tools in understanding this system’s functional anatomy.

We describe and implement a workflow for clustering thousands of genes across thousands of olfactory voxels in the ABA, in turn discovering sparse and novel “genomic signatures” for the early olfactory system’s constituent structures. Our analysis both recapitulates well-known subdivisions and sub-systems within olfaction, and also identifies novel, candidate subregions and corresponding enriched gene-sets that merit further experimental investigation. The tools described here may be an important complement to ongoing efforts to map and characterize olfactory subsystems.

## Materials and methods

### Data

All data were obtained from the Allen Brain Institute’s (ABI) mouse brain expression project (www.brain-map.org), which comprises a set of in-situ hybridization (ISH) experiments cataloging expression of the entire mammalian genome across the C57BL/6 (P56) mouse brain, at cellular resolution. The data resources of the ABI are documented amply in other publications [3,4] as well as in ABI whitepapers (http://help.brain-map.org/display/mousebrain/Documentation). Briefly, ABI data are available as raw ISH image files, and also as a registered 3D atlas (voxel size: 200 μm x 200 μm ^x^ 200 μm) in which each atlas voxel is a vector quantifying expression energy across the genome, at a particular location in the brain. The olfactory bulb comprises 990 such voxels in this data set. In the present study, we used only the registered data, and investigated a quality-controlled subset of genes (3,041 genes out of ~20,000) that has been used in several previous publications [15,16]. This set represents genes showing strong correlation across the coronal and saggital image series of the Allen Brain Expression Atlas.

The complete set of genes we used (3,041 of them) was culled over several publications. The first draft coarse atlas was obtained on 25 micron thick sagittal brain sections taken every 200 microns, for a total of ~20 sections for a single hemisphere [3] From these data, a subset of 4,376 genes of ‘neurobiological interest’ (described in [17]) were chosen for a more comprehensive and anatomically more granular analysis. While this is evidently a 4-to-5 fold undersampling of the complete genome, it is worth noting that a large fraction of the genome (~10%) is not expressed in the brain, and an additional ~20% is only negligibly expressed [3]. Of the ~ 80% of genes expressed above background, a sizeable fraction (~37%) showed “near ubiquitous” expression, meaning that these genes showed little difference in expression across cell types. Indeed, gene ontological (GO) analyses of the set of near ubiquitous genes was consistent with their involvement in basal cellular function (cellular metabolism, protein metabolism, basal/homeostatic gene function, etc).

For each of the ~4,000 ‘genes of interest’ from the sagittal series described above, additional ISH experiments were done, taking coronal sections, also every 200 microns, for a total of ~56 sections (per gene). From these ~4000 genes, a “higher consistency” [15] subset was obtained that exhibited high expression concordance between coronal and sagittal data sets. The Pearson correlation coefficient was calculated between voxels of the sagittal and coronal data sets for each gene, and those genes in the lowest quartile of the spatial correlation coefficient distribution were discarded. The remaining 75% of genes are those that make up the set of 3,041 genes used in the present study. This set of genes is thus a representative and relatively unbiased subset of “neurobiologically interesting” genes whose expression is consistent across experiments. According to the Allen Brain Institute’s release and publication [18] of the Anatomic Gene Expression Atlas (AGEA) data, these genes are strongly enriched in “[GO] categories related to neuronal cell process and function, GABA receptor activity, ion and potassium channel binding, neuron differentiation, axon guidance, synaptic transmission and long-term potentiation.” This specific gene set has been used in other publications [15,16,19]. In some analyses, we extracted smaller subsets of ‘physiological genes’, which consisted of those genes listed in the IUPHAR database () of ion channel genes [20] (146/3041 genes).

### Data handling, dimensionality reduction and clustering

All data analysis was performed in Matlab (Mathworks). Several routines from the ‘Brain Gene Expression Analysis’ toolbox, written by Grange et al [21], were modified for low-level data handling (extracting matrix columns corresponding to named brain regions, etc). All other analysis functions (dimensionality reduction, clustering, etc) were custom-written, and are available at https://github.com/CastroLab.

Our clustering framework employed non-negative matrix factorization [22,23], in which a low (s) dimensional approximation of a matrix, A, is sought taking the form:

A=WH (1)

Where A is an m x n data matrix, and W and H are m x s and s x n matrices containing feature vectors and their weights, respectively. We performed the factorization using the standard Matlab implementation (nnmf.m), using the alternating least-squares algorithm initially proposed by [22]. Briefly, this algorithm iterates over the following steps:

1. assume W is known and solve the least squares problem for H using:
  $$(W^{\text{T}}W)H=W^{\text{T}}A$$ (2)
2. set negative elements of **H** → 0
3. assume H is known and solve the least squares problem for W using:
  $$\left(HH^{\text{T}}\right)W^{\text{T}}=HA^{\text{T}}$$ (3)
4. set negative elements of **W→0**

We used 1000 iterations as our stopping criterion for the maximum number of iterations.

## Results

### Summary of workflow

**Fig 1:**
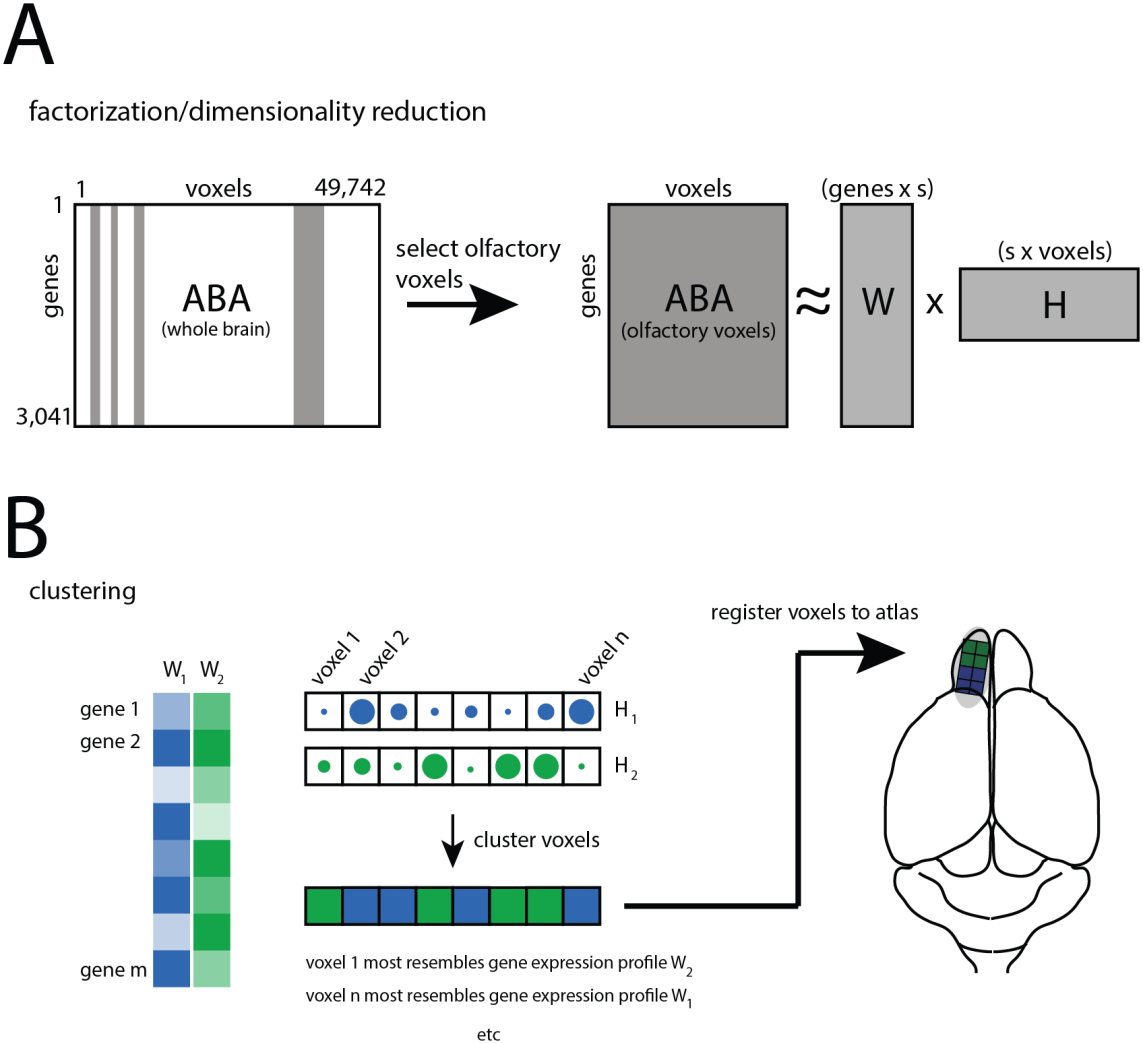
Workflow. **A)** The principal data set was a genes x voxels matrix [3,21] cataloging brain-wide expression. Columns corresponding to known olfactory areas were selected, and the dimensionality of the resultant sub-matrix was reduced using non-negative matrix factorization (NMF). The cartoon shows an example of reducing the original data from m-dimensional to s-dimensional, where m>>s. **B)** NMF yields a new basis set (column vectors) as well as weights of voxels in the new basis (row vectors). Voxels are readily clustered by selecting the largest weight, and registration of clustered voxels to the anatomical atlas reveals the spatial patterning of clusters. Note that the clustering itself is blind to spatial relationships among voxels. The cartoon shows an example where s = 2.

Our overall goal was to identify the olfactory system’s molecular subdivisions, as specified by regional differences in gene correlations across thousands of genes. To do this, we developed a gene-clustering and dimensionality reduction workflow based on non-negative matrix factorization (NMF) [23](Fig 1). NMF-based methods have enjoyed frequent use in gene-clustering applications owing to the intrinsic non-negativity of expression data, and the ready interpretability of NMF-derived expression profiles. Intuitively, NMF discovers from data a handful of highly informative ‘signature’ expression profiles (formally termed a basis set) that summarize a large fraction of total variability in gene expression. Brain areas can then be readily clustered by their resemblance to a given one of these expression profiles (formally, their ‘weights’ in the new basis set). Notably, this decomposition into signature expression profiles can be done at arbitrary levels of granularity. Recent investigations of genomic anatomy in the hippocampus [5] and cortex [4] have used methods similar to ours.

The essential components of our workflow are illustrated in Fig 1. Briefly, the full data set (the matrix labeled ‘ABA’ in Fig 1) was a 3,041 x 49,742 matrix of genes x voxels cataloging gene expression across the entire mouse brain. Each voxel (i.e. each column of the matrix) catalogs the expression of thousands of genes, and was obtained by analyzing thousands of in-situ-hybridization (ISH) experiments, and spatially registering these data to a common 3D grid (the Atlas proper) with 200 cubic-micron voxels. This matrix catalogs ~ 15% of the entire genome (~20,000 genes), and is a previously published, quality controlled subset of the ABA for genes exhibiting highly correlated expression between both coronal and saggital ISH experiments [3,21]. Although contiguous columns of this matrix tend to represent spatially contiguous voxels, coordinates in anatomical space are not formally represented in column ordering. Indeed, it is important to emphasize that all matrix operations are performed in ‘gene space’, and blind to relative spatial positions. Visualization of voxels in their correct spatial context was achieved through separate lookup tables that assign voxels to grid coordinates.

We first extracted a sub-matrix of 2,310 columns corresponding to all olfactory areas, and performed NMF (see Methods) to approximate this as the product of a matrix of basis vectors (W) and matrix of corresponding weights (H) (Fig 1A). This was done for subspace sizes (s) ranging from 2 to 7. Owing to the inherent sparseness of NMF decompositions [23], clustering (Fig 1B) was readily performed by selecting the largest resultant weight for each voxel. Clustered voxels were then registered back to the Atlas to visualize their spatial relationships.

### Genomic definitions of gross olfactory system subdivisions

**Fig 2:**
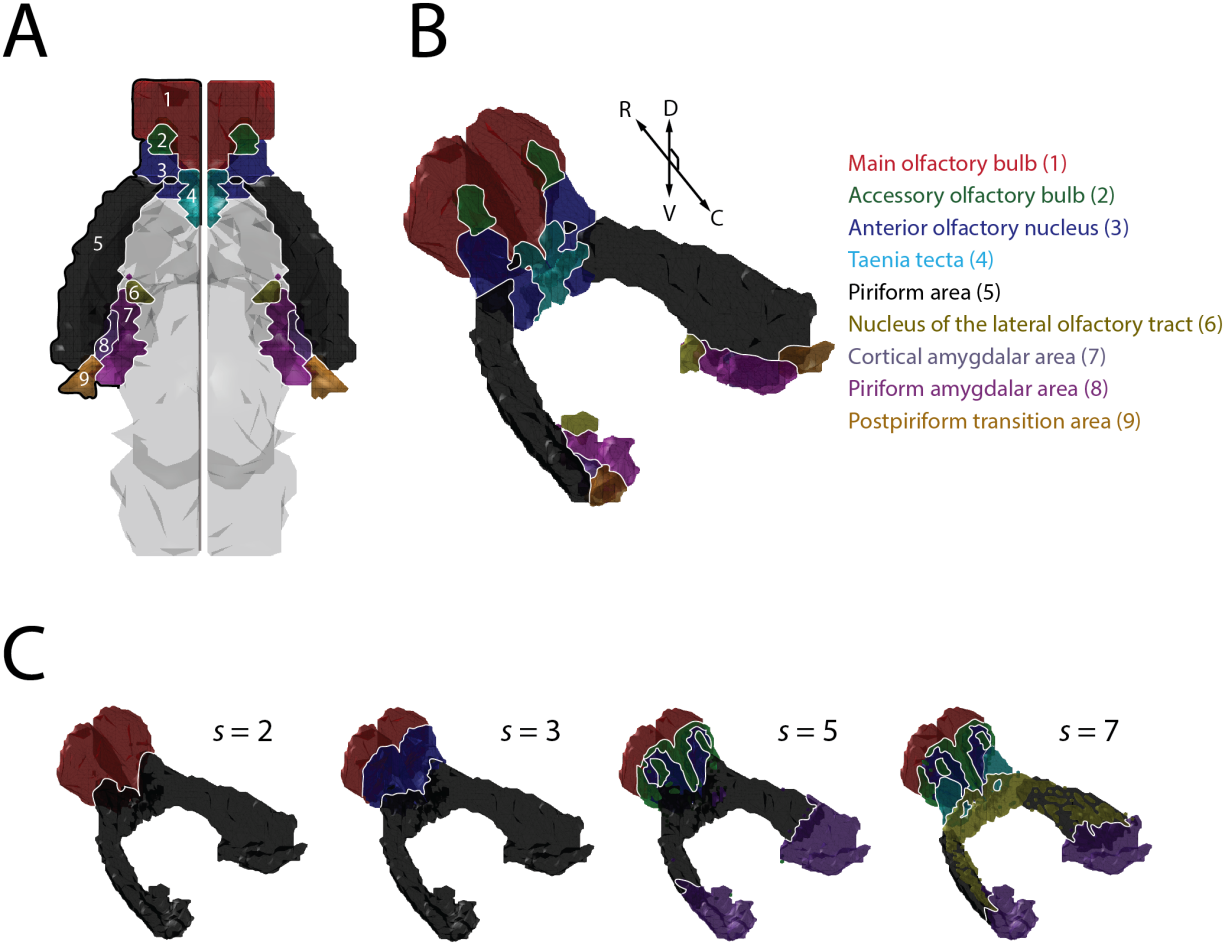
Genomically-defined divisions of the olfactory system. **A)** Horizontal-plane projection of the mouse olfactory system as demarcated in the Allen Brain Atlas (named subregions shown to the right). Diencephalic, midbrain, and brainstem structures are shown in gray for spatial context, though voxels comprising these structures were not used in the analysis. Abbreviations: R-rostral, C-caudal, D-dorsal, V-ventral) **B)** 3D rendering of the olfactory system, showing its various subdivisions, as in (A). The main (1) and accessory (2) olfactory bulbs define the rostral-most pole of the mouse brain and receive direct input from the olfactory periphery; the remaining structures receive both direct and indirect bulbar input, and lie along the brain’s ventral surface. **C)** Non-Negative Matrix Factorization (NMF)-based clustering of the olfactory system, for various choices of subspace size, s (see text for details).

Applying the framework described above, we decomposed the olfactory system into its genomically defined subdivisions, at several levels of granularity. Fig 2A and 2B show the olfactory system’s major, classically defined *anatomical* subdivisions, for the ABA’s most granular available taxonomy. Fig 2C shows several *genomic* parcellations of the olfactory system, for NMF decompositions at several levels of granularity (subspace size = s). For a choice of s = 2, the olfactory system was clearly partitioned into its major sensory recipient (bulbar) and higher cortical subdivisions. At choices of s = 3 and s = 5, the decomposition began to reveal substructure within the anterior and posterior subdivisions of the olfactory bulb, as well as clearly demarcating the limbic (amygdala-associated) divisions of olfactory cortex. For the most granular decompositions we performed (s = 5 and s = 7), the olfactory system was partitioned into subdivisions that recapitulated many well-known anatomical subdivisions, including the accessory olfactory bulb (AOB), the anterior olfactory nucleus (AON), the amygdalar portions of olfactory cortex, and the anterior and posterior subdivisions of piriform cortex.

**Fig 3:**
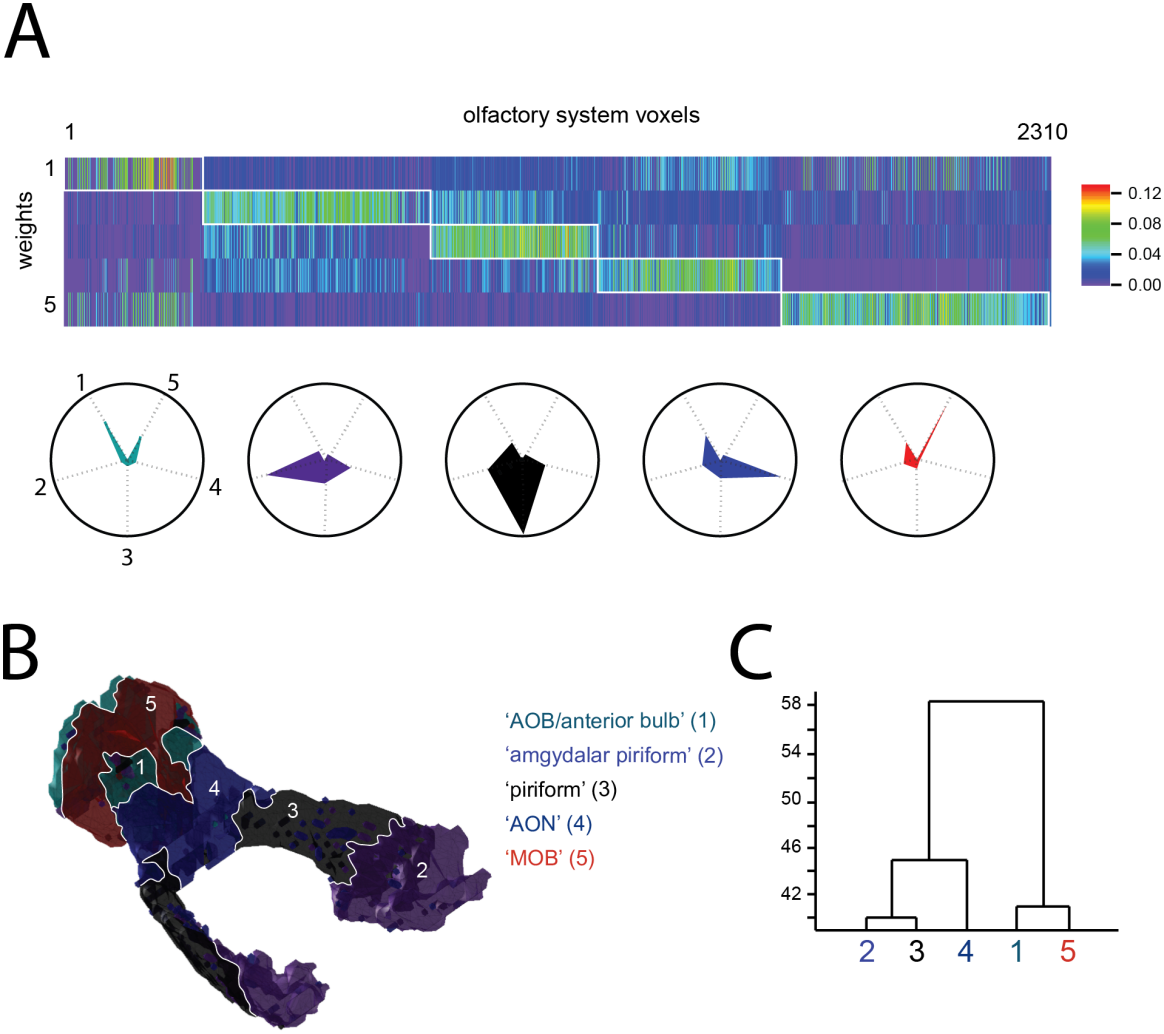
Olfactory system subdivisions defined by ‘physiological’ genes. **A)** Top: matrix of weights (H matrix) for all olfactory system voxels, for a 5 dimensional NFM decomposition. Columns have been sorted by peak value (preserving the order and values of row-contents) to reveal the block-diagonal structure of the matrix. Bottom: Polar plots of the average column-vector for each diagonal block, showing categorical assignment of voxels to one the five weight dimensions, to the relative exclusion of others. **B)** Spatial arrangement of clustered voxels shown in (A), in Brain-Atlas coordinates. Note the strong spatial contiguity of most of the clusters (with region 1 being a notable exception). Colors correspond to the polar plot colors in (A). Region names in quotes are given as a heuristic summary—they do not necessarily align with ABA or other atlas definitions. **C)** Dendrogram showing inter-cluster distances (Euclidian distance between basis vectors).

The brain’s ‘components’ can be defined by a variety of molecular criteria, including the patterned expression of developmental markers, transcription factors, ion channel genes, genes for the synthesis and loading of neurotransmitter, as well as many others. To develop a ‘functional’ parcellation of the olfactory system—that is, one that is likely to reflect differences in biophysical and synaptic properties—we performed an additional analysis using a more constrained set of physiologically relevant genes. Specifically, we performed the NMF decomposition for the subset of genes (143 of the 3,041) listed in the IUPHAR database [20] corresponding to voltage or ligand-gated ion channels, and G protein coupled receptors (GPCRs). The results of this decomposition are shown (for a choice of s = 5) in Fig 3. Fig 3A shows the resultant matrix of weights, H (see workflow in Fig 1), and its block-diagonal structure highlights the ‘categorical’ nature of NMF-derived representations. Briefly, a given voxel tends to be well-characterized by a single basis vector, to the relative exclusion of others.

**Fig 4:**
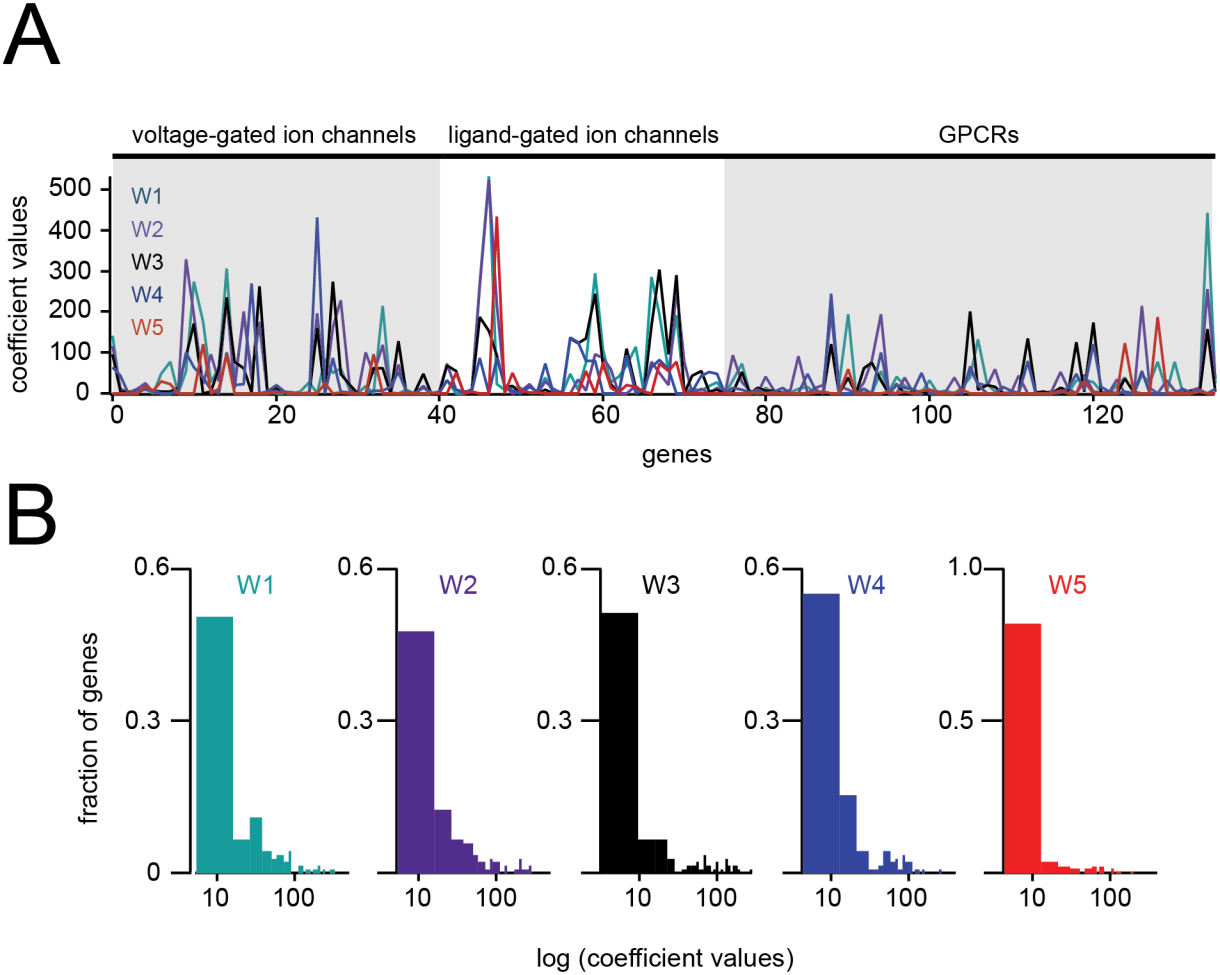
Gene expression profiles derived from NMF are sparse. **A)** Expression profiles (the basis vectors W1-W5) for the three families of IUPHAR genes investigated (see text). **B)** Histograms of each expression profile. The x-axis is on a log-scale to reveal structure (gene ‘hits’) in the distribution tails that is not otherwise evident.

The spatial organization of these ‘physiological’ clusters is illustrated in Fig 3B, and these map on well to several of the olfactory system’s known anatomical subdivisions (compare to Fig 2). While these clusters and their spatial relationships are in themselves interesting, their greater potential utility comes from the fact that each cluster is defined by a unique and characteristic signature expression profile, discovered through dimensionality reduction. Fig 4A shows the five signature profiles for the decomposition described above. Note that these profiles tend to be sparse: there are only strong ‘hits’ for a small subset of genes, with the great majority of genes contributing little to nothing to the definition of a given cluster. The sparseness of the expression profiles is also illustrated and quantified in the histograms (Fig 4B).

### Validating olfactory subdivisions, and discovering candidate subdivisions using the ABA

The parcellations described above recapitulate many of the major known olfactory structures and subregions. While these are important for identifying sets of key differentiating molecules for major structures, it is also potentially interesting to investigate more spatially granular parcellations of the olfactory bulb (OB) in an exploratory context. Understanding the degree to which the OB is molecularly heterogeneous and modular vs homogeneous and ‘equipotential’ could help constrain models of early olfactory function. Specifically, evidence of genetic modularity and heterogeneity across the bulb would be consistent with current proposals that the OB is a collection of specialized, parallel modules [10,24], and not a single ‘multipurpose’ columnar circuit simply iterated many times in parallel.

To ask whether our methods are in principle capable of making these more granular distinctions, we first investigated olfactory bulb subsystem organization in a case where we expected to find it: between the anterior and posterior divisions of the accessory olfactory bulb (AOB)—a small, well delineated olfactory subsystem that processes non-volatile compounds, and makes critical contributions to many innate behaviors [25,26]. The AOB’s two divisions are dissociable on the basis of their unique inputs from different receptor classes [27–29], their distinct central projections, and their differential expression of lectins and developmental control molecules [30–32].

**Fig 5:**
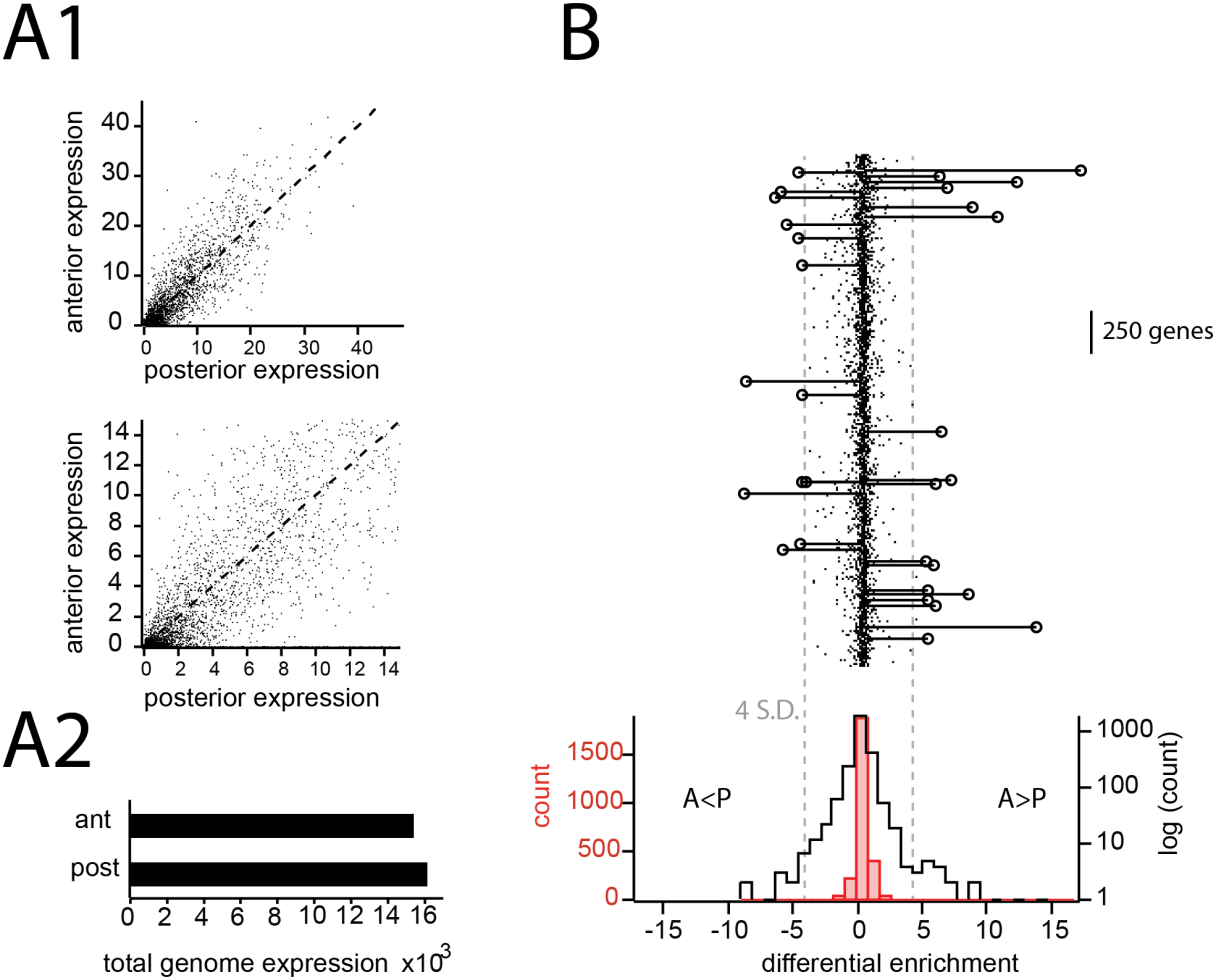
Gene expression and differential enrichment in the anterior vs posterior AOB. A) 1: Scatter plots of gene expression for all 3,041 genes in the anterior vs. posterior AOB. Each point is an average across anterior voxels and posterior voxels (see Methods). Dotted line indicates equal expression in the anterior and posterior. Lower graph is the same data on an expanded scale. A2) total expression of all genes in the Anterior AOB and posterior AOB, illustrating no notable difference between the two. B) top. plot of differential enrichment (anterior AOB enrichment—posterior AOB enrichment; see text) for all genes. The majority of genes were symmetrically or near-symmetrically expressed in the anterior and posterior AOB. Genes with asymmetry scores exceeding 4 standard deviations are shown in lines and markers. The remainder of genes are shown as dots. Bottom: Histogram differential enrichment, shown in both linear (red) and log axes.

Inspecting and quantifying expression between the anterior and posterior halves of the AOB (Fig 5A and 5B), it was evident that, on average, genes were symmetrically distributed across this structure’s midline axis. For each of the 3,041 ABA genes, we also calculated a differential enrichment score (see Methods), which quantified expression asymmetry across the A-P axis. A histogram of these scores across genes was sharply peaked at 0 (symmetric expression), but a population of highly differentially enriched genes could be observed in the tails of this distribution.

**Fig 6:**
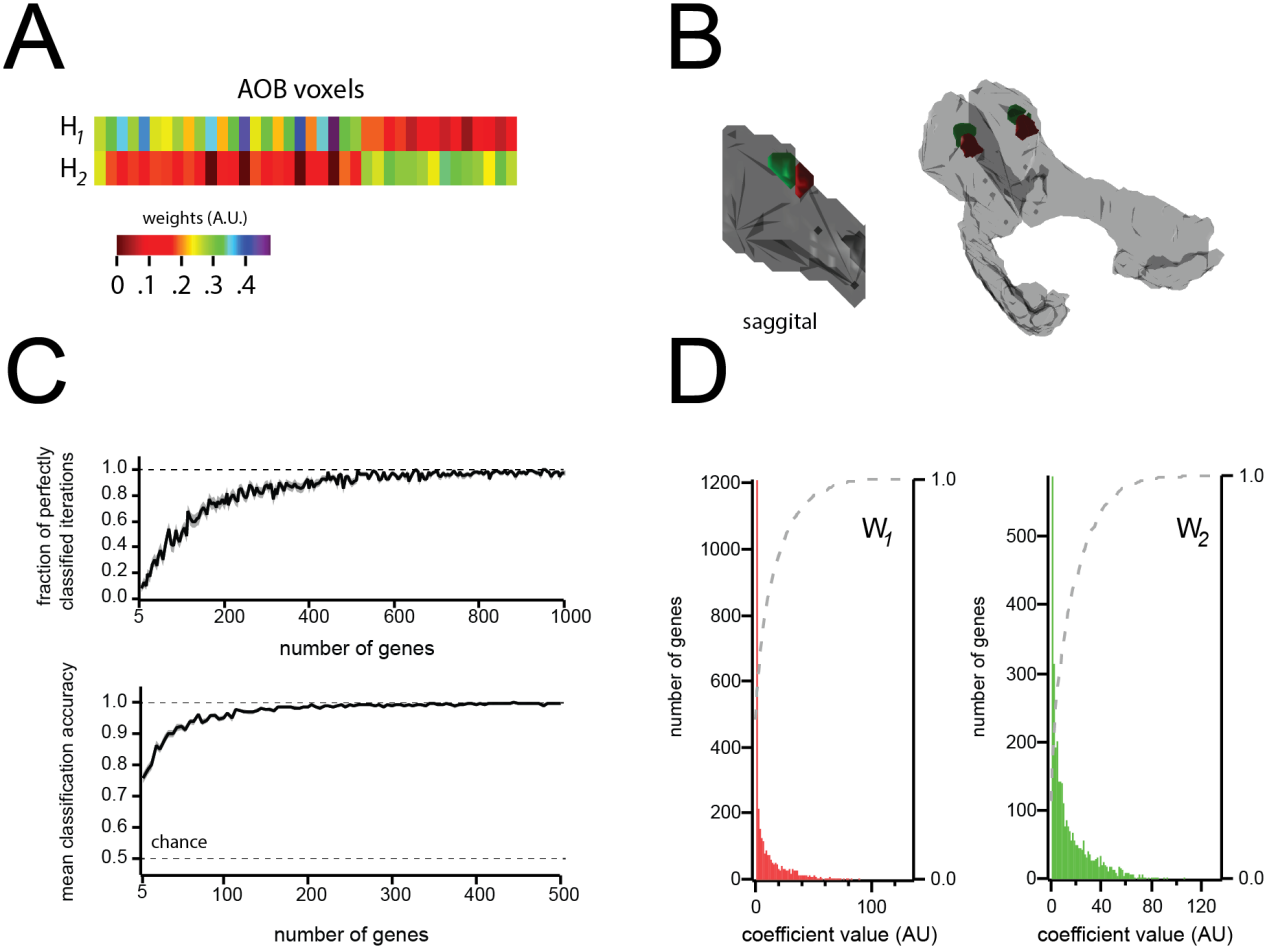
Clustering (s = 2) returns the known anterior and posterior sub-divisions of the AOB. **A)** Weights matrix (See Fig 1) showing dichotomous segregation of AOB voxels. **B)** NMF reveals spatially contiguous subregions of the AOB clearly corresponding to the structure’s (known) anterior and posterior sub-divisions. **C)** classification accuracy of NMF vs number of genes included. *Top*: fraction of ‘perfectly classified voxels’, as a function of number of genes (see Methods). *Bottom*: Mean classification accuracy vs. number of genes used for the factorization. **D)** Histogram of basis-vector values, illustraing sparseness of the basis vectors (i.e. a small number of genes defines membership in anterior vs. posterior).

Interestingly, when we clustered AOB voxels using a 2 dimensional factorization (see Methods), the two resultant clusters clearly corresponded to the structure’s anterior and posterior halves (Fig 6). As with the factorizations described above, the expression profiles for these clusters were sparse, with relatively small numbers of genes contributing to the definitions of the AOB’s anterior and posterior halves.

**Fig 7:**
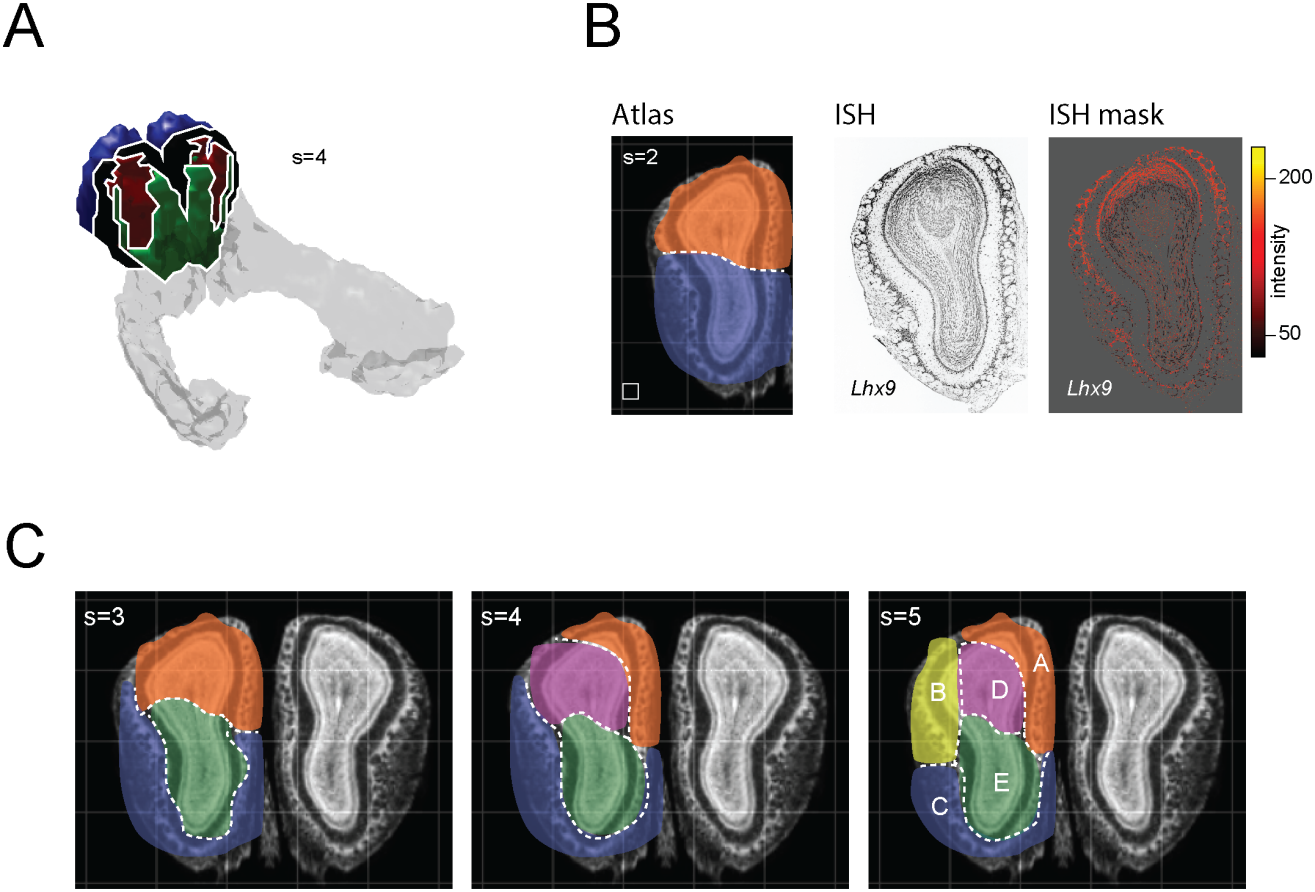
Clustering the main olfactory bulb reveals candidate genomic subdivisions. **A)** Location of NMF-derived clusters (s = 2) in ABA coordinates (see S1 File for genes comprising the clusters). **B)** left: NMF-decomposition (s = 2) of the fifth coronal section of the expression atlas reveals clear dorsal v. ventral domains (white box indicates ABA voxel-size); middle: ISH image from the ABA of *Lhx9* –the highest ranked gene in the dorsal cluster; right: segmented expression mask showing strong dorsal pattering of Lhx9 (contrast unadjusted from the raw, downloaded mask). **C)** NMF decompositions of the OB (5^th^ coronal section) at progressively greater granularity, revealing the ventral glomeruli as a clear and contiguous genomic territory as well as two distinct domains within the dorsal glomeruli. The lettering of clusters in the s = 5 case corresponds to the lettering in Fig 8. See supplementary info (S1 File) for genes comprising each cluster.

Given our methods’ successful identification of granular substructure within the AOB (a ‘validatory’ case), we next derived a candidate parcellation of the main olfactory bulb (an ‘exploratory’ case). Recent work supports the idea that bulb is comprised of molecularly and behaviorally specialized subsystems [10,14,33–35], and understanding these at the scale of the genome could provide important clues about their local computations and potential functional roles. Fig 7A shows a 4-dimensional decomposition of the olfactory bulb (with the AOB excluded), illustrating that the bulb can be parceled into molecularly distinct territories on the basis of spatial correlations between genes. The rank-ordered genes comprising each of these territories can be found in the supplemental information (S1 File).

While this analysis revealed large, spatially contiguous clusters along the rostro-caudal extent of the bulb, it was generally difficult to map these clusters on to distinct lamina and known bulbar domains. To get a clearer portrait of how and whether clusters corresponded to histological layers, we applied our workflow on a single (2D) coronal section of the bulb (specifically, the 5^th^ coronal section of the reference atlas) (Fig 7B and 7C). For a choice of s = 2, the bulb was clearly segregated into broad dorsal and ventral domains, and inspection of the ISH data from the leading hit from the dorsal domain revealed a marked enrichment of the LIM homeodomain transcription factor *Lhx9* in the bulb’s dorsal aspect (Fig 7B). This is, to our knowledge, the first report of strong dorsal patterning of this gene in the bulb.

**Fig 8:**
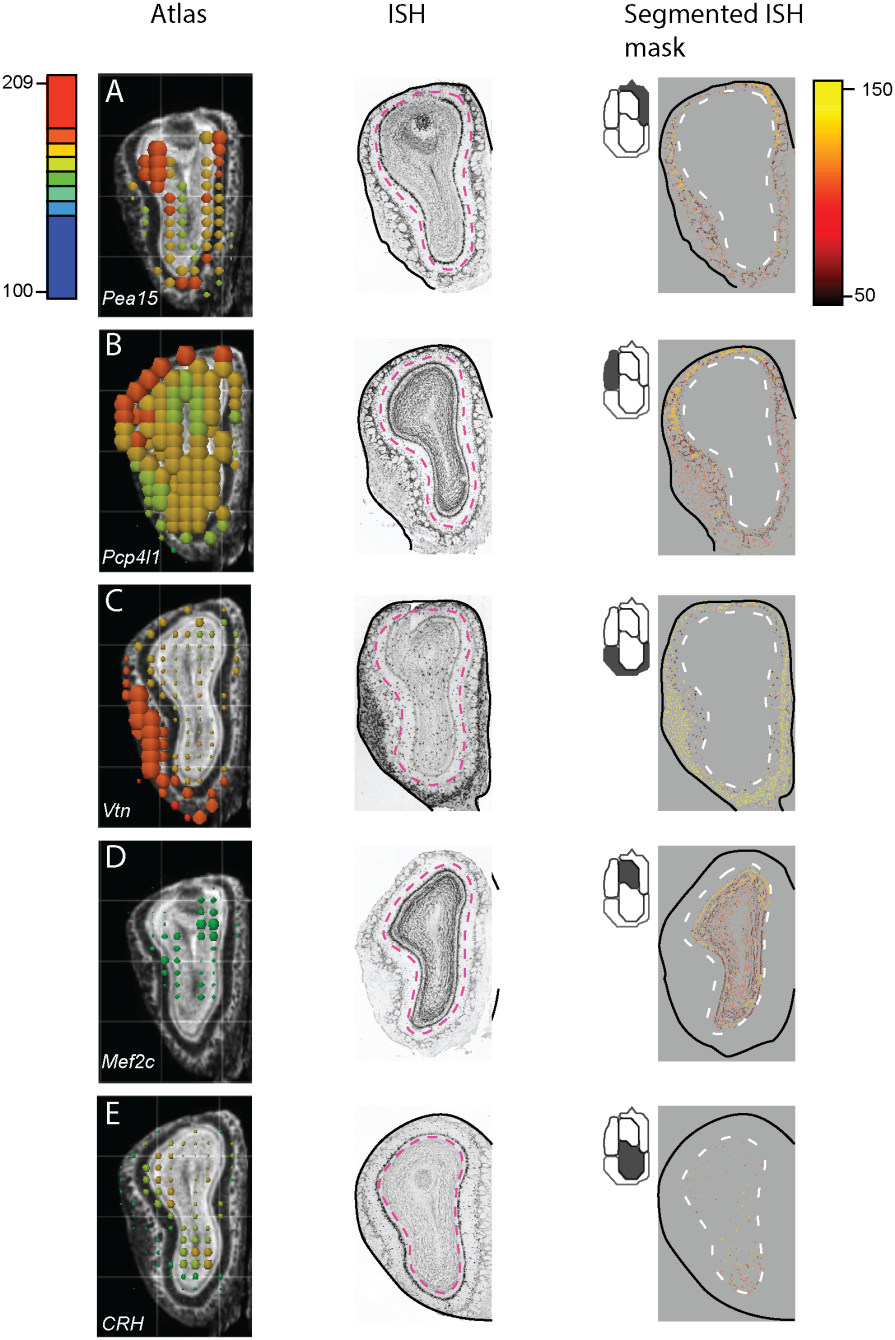
Expression patterns of leading genes in each of the 5 NMF-derived OB clusters. Letters (A-E) correspond to clusters labeled in Fig 7C. Icons at the top-left of the segmented masks show which cluster is being shown. **A)** Phosphoprotein enriched in astrocytes 15 (Pea15); rank # 2/3,041; enriched in the dorsomedial glomerular cluster. **B)** Purkinje cell protein 4-like (Pcp4l1); rank #2/3,04; enriched in the dorsolateral glomerular cluster. **C)** Vitronectin (Vtn); rank #3/3,041; enriched in the ventral glomerular cluster. **D)** Myocyte enhancer factor 2C (Mef2c); rank #7/3,041 (#3 of protein coding genes); enriched in dorsal mitral and granule cells; **E)** Corticotropin releasing hormone (CRH); rank #24/3,041; enriched in ventral granule cell population.

As the number of clusters was increased from s = 3 to s = 5, the bulb was partitioned into subregions that revealed distinct glomerular domains. For the choice of s = 5, we observed a cluster corresponding to the ventral glomeruli, and two separate clusters demarcating the dorsomedial vs. dorsolateral glomeruli. The remaining 2 clusters evidently demarcated the dorsal vs. ventral granule cell and mitral cell layers. The separation of the dorsal glomeruli was especially interesting, as these may map onto the two dorsal domains defined by the projection patterns of Class I and Class II OSNs [36]. Fig 8 shows several examples of raw in-situ data and expression masks for leading genes in each of the five identified clusters (these are also described more in the discussion section). The full list of rank-ordered genes in each of these clusters is available in the supplemental information (S1 File).

## Discussion

Using the resources of the Allen Brain Atlas, we have investigated the molecular anatomy of the olfactory system at genomic scale. We described data-driven parcellations of various olfactory sub-regions that capture spatial correlations across thousands of genes. In doing this, we also derived ‘signature’ gene expression profiles that are characteristic of different subregions. Our analysis recapitulates many of the fundamental olfactory sub-regions familiar from classical anatomy, and also points to the existence of candidate sub-regions (and their most characteristic genes) within the olfactory bulb. These enriched gene sets can immediately form the basis for targeted, follow-up experiments to investigate candidate modules within the bulb. In future studies it will also be important to explicitly test for functional differences between these modules, as differences in expression do not necessarily manifest as differences in excitability or synaptic function.

Several other studies have used the resources of the Allen Brain Institute to investigate the ‘genomic anatomy’ of CNS’s major phylogenetic subdivisions [3], as well as of specific structures including the hippocampus and neocortex [4,5]. Similar to the results we obtained, these studies derived molecular, data-driven parcellations of brain structures, in some cases recapitulating nuclear and laminar structures originally identified using classical histological methods. Among these model anatomical systems, the OB has several virtues that make it attractive for follow-up genomic/anatomic investigation. The principal of these is the well-known and well-characterized topography and modularity of the OB’s sensory inputs. At coarse scales, the OB surface can be partitioned into a handful of non-overlapping territories defined by the stereotyped axonal projections of olfactory sensory neurons with common signal transduction pathways[11,14,37]. Superimposed on this large-scale “zonal” map is a more granular and densely tiled map defined by the bulb’s numerous glomeruli—regions of dense neuropil that each receive convergent inputs from idiotypic sensory neurons [12,38]. While a considerable amount of recent work has shown that these parallel systems in some cases respond to different characteristic ligands and support distinct behaviors, it is largely unknown whether there is heterogeneous ‘readout’ of these inputs by specialized intrinsic circuits of the OB. At one extreme, the OB’s principal neurons may comprise a molecularly homogeneous group that processes information similarly across inputs. At the other extreme, these principal neurons may be molecularly diverse and/or organized into several classes to support input-specific processing. Our results preliminarily support the latter view, and suggest that the OB is comprised of at least several genomically distinct territories. Additional work will be needed to determine whether these regions are functionally distinct as well.

One especially intriguing finding was our observation of three genomically distinct, spatially non-overlapping glomerular domains. For a choice of 2 clusters, the bulb was partitioned into dorsal vs. ventral subdivisions, and with increasing granularity of clustering the glomerular layer was partitioned into ventral and dorsomedial domains. For the s = 3 and s = 4 decomposition (Fig 7C), the ventral glomeruli extended more dorsally along their lateral aspect, in a manner consistent with glomerular subdivisions demarcated by OCAM [11,39]. For the s = 5 decomposition, two dorsal glomeruli were partitioned into medial and lateral subdivisions, reminiscent of the two separate dorsal domains targeted by Class I and Class II ORNs [36].

Although our analysis has generated large numbers of candidate genes for future experimental inquiry (the set of all rank-ordered genes, by cluster, is provided in S1 File), several specific cases are worth briefly summarizing. First, we note that our identification of vitronectin—an extracellular matrix component—as a marker of the ventral glomerular cluster (ranked #3, Fig 8C) is consistent with results reported in the microarray study by Lin et al [40] (ranked # 13 in that study). Given the concordance between these two large-scale screens, and the known role of vitronectin in regulating neurite outgrowth and neuronal migration, the role of this gene in specifying glomerular topography may merit further investigation. Second, we found that a leading marker (rank #2) of the dorsolateral glomerular cluster was the gene Pcp4l, which has been shown in other studies to be differentially expressed within the OB in a manner regulated by cAMP concentrations [41]. This same gene is also one of only 8 mRNAs (in a genome-wide study) showing activity dependent regulation in mouse OSNs following naris occlusion. Finally, we observed that the LIM homeodomain transcription factor Lhx9 was the highest-ranked gene defining the bulb’s dorsal aspect (Fig 7B), and correspondingly was strongly and selectively enriched in dorsal glomeruli, mitral cells, and granule cells. Other Lhx genes have been shown to be important for OSN development (Lhx2)[42] and the genetic specification of innate olfactory behaviors (Lhx6)[43]; Lhx9 may therefore be an interesting candidate for delineating a topographically defined olfactory sub-system.

Although our study is novel in investigating olfactory circuit organization at the scale of the genome, it is important to also summarize its limitations. First, because the ABA ISH data are compiled across experimental subjects, we do not have information about co-expression of molecules at the single cell level. Our analysis can only report on comparatively large-scale ‘zonal’ molecular heterogeneity across the olfactory system and olfactory bulb. Similarly, the spatial granularity of our analysis is ultimately limited by the voxel size of the ABA, which is large (200 μm on a side) relative to single neurons and neuronal microcircuits. While it would be interesting to systematically study potential molecular variability within and between cell types, and across glomerular columns, this is likely not possible with the registered ABA data.

In sum, we have developed and implemented methods for studying the large scale genomic anatomy of the early olfactory system. The parcellations we derive using these methods identify both known and novel subregions of the olfactory bulb, and the molecular markers identified from this can serve as the starting point for future inquiry into the subsystem organization of olfaction.

## Supporting information

S1 File Excel spreadsheet with rank-ordered genes by cluster, and corresponding NMF coefficient values. (XLSX)
